# Genetic Evidence of Multiple and Diverse Range Expansion Events From an Outbreak of the Crown‐of‐Thorns Seastar, *Acanthaster* Cf. *Solaris* on a Subtropical Reef

**DOI:** 10.1002/ece3.72799

**Published:** 2025-12-22

**Authors:** Matt J. Nimbs, Maria Byrne, Steven J. Dalton, David Maguire, Hamish A. Malcolm, Nicole Strehling, Brigitte Sommer, Melinda A. Coleman

**Affiliations:** ^1^ Fisheries Research, New South Wales Department of Primary Industries and Regional Development National Marine Science Centre Coffs Harbour New South Wales Australia; ^2^ National Marine Science Centre Southern Cross University Coffs Harbour New South Wales Australia; ^3^ School of Life and Environmental Sciences The University of Sydney Sydney New South Wales Australia; ^4^ Fisheries Management, Fisheries and Forestry NSW Department of Primary Industries and Regional Development Coffs Harbour New South Wales Australia; ^5^ Marine Operations, Aboriginal Fishing and Marine Conservation NSW Department of Primary Industries and Regional Development New South Wales Australia

**Keywords:** eastern Australia, marine range expansion, mitochondrial genetic connectivity, tropicalisation

## Abstract

Understanding the dynamics of range expanding species, particularly those that have effects on recipient reefs, is vital to inform management and conservation strategies. A rare outbreak of the tropical Crown of Thorns seastar (*Acanthaster* cf. *solaris*) (COTS) on a subtropical coral reef and a subsequent control program, presented the opportunity to determine the likely origins and number of recruitment events that may have generated this outbreak. The presence of both cosmopolitan and regional mitochondrial haplotypes in this outbreak population indicated larval connectivity via the poleward flowing East Australian Current (EAC) from the Great Barrier Reef (GBR) and Coral Sea. High genetic connectivity with the GBR, based on mitochondrial COI variation, where multiple COTS outbreak events have occurred through time, suggests a high risk of future outbreaks in subtropical eastern Australia. Additionally, levels of COI genetic diversity in the subtropical outbreak population are consistent with either ongoing recruitment or multiple source populations, rather than a new founder population from a single dispersal event. Outbreaks of COTS are likely to become a more frequent occurrence under future climate change scenarios as the poleward‐flowing EAC strengthens and brings warmer waters to subtropical reefs. Agencies that manage subtropical reefs should prepare and plan for future outbreak events and develop policy and management strategies for this range extending species.

## Graphical abstract

This study examines the population genetics of a Crown of Thorns seastar (Acanthaster cf. solaris) outbreak on a subtropical coral reef. We demonstrate that this population originated from larval connectivity via the East Australian Current from the Great Barrier Reef, with high genomic connectivity indicating substantial risk of future outbreaks as ocean currents strengthen under climate change.

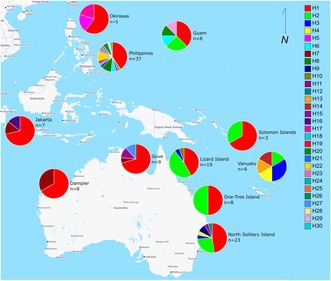

## 1 Introduction

Tropicalisation of temperate reefs; the range expansion of tropical species into historically temperate waters necessitates new policy to guide management and response interventions (Vergés et al. 2014). As novel species arrive and persist, marine managers must decide whether to take action to control these incursions in efforts to maintain current ecosystem states or accept new ecological communities that are different to those they currently manage (McGeoch et al. 2024) that may be perceived as a ‘natural’ state. Although redistribution of taxa such as corals or harvested species into temperate waters is often seen as desirable, the arrival of species that can change ecosystems can warrant control programs to slow incursions (e.g., sea urchins (Watanabe and Harrold 1991; Westcott et al. 2022) and *Drupella* snails (Bessey et al. 2018; Zhang et al. 2024)). Central to informing such decisions and the urgency for action, is understanding the origin, dispersal pathways and frequency of incursion of range expanding, destructive species.

Subtropical reefs are biodiversity hotspots that are located in the biogeographic transition between tropical, subtropical and temperate marine environments housing unique overlapping assemblages of tropical and temperate species. These regions also serve as important climate refugia for tropical species shifting poleward due to ocean warming and the strengthening of currents (Dalton et al. 2020; Malcolm et al. 2011). They can also house unique and endemic coral species (González‐Pech et al. 2022; Veron et al. 1974). A particularly destructive natural threat to subtropical reefs are outbreaks of the corallivorous crown‐of‐thorns seastar (COTS) *Acanthaster* spp. (Babcock et al. 2016; Deaker and Byrne 2022). COTS are significant predators of coral and can inflict considerable, long‐lasting damage to reefs when their populations boom (outbreaks), leading to high levels of coral death (Foo et al. 2024; Pratchett et al. 2017). Repetitive outbreaks are a major cause of coral reef degradation (Babcock et al. 2020; Endean 1982; Vercelloni et al. 2017; Wooldridge and Brodie 2015) and large control programs to limit their negative effects exist for many coral reef systems globally (Matthews et al. 2024; Woerheide et al. 2022; Yamaguchi 1986).

Poleward range shifts of COTS into subtropical reefs are facilitated by their high fecundity (Deaker and Byrne 2022), long‐distance dispersal and long pelagic larval duration (several weeks) combined with a broad thermal window for larval development (25°C–31.6°C) (Johnson and Babcock 1994; Lamare et al. 2014). As a result, warming waters are now facilitating COTS larval recruitment to cooler, higher latitude reefs globally (Yasuda 2018).

**FIGURE 1:**
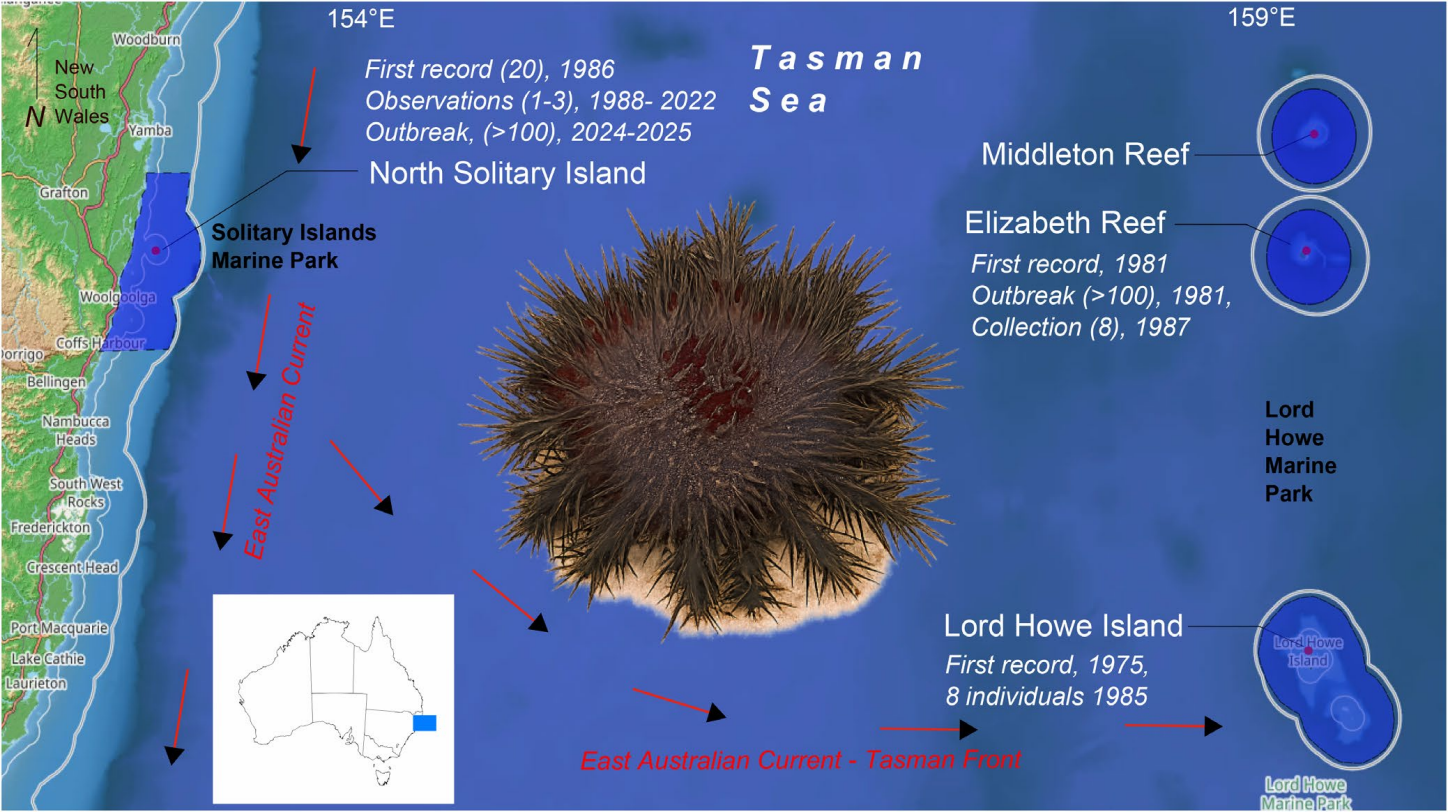
Map of subtropical reefs in New South Wales (coastal and oceanic) and their history of COTS observations. Position and flow of the East Australian Current and Tasman Front marked as red arrows. Marine Protected areas in dark blue. Refer to Table 1 for observation sources. Map data from OpenStreetMap, https://www.openstreetmap.org/copyright. Western Pacific *Acanthaster* cf. *solaris* image (edited from original) used with permission from MikeMules https://www.inaturalist.org/photos/345589503.

On Australia's east coast, the geographic range of COTS outbreaks has expanded poleward onto oceanic reefs and into coastal areas over the past 50 years (Sommer et al. 2025) (Figure 1). Despite the very patchy distribution of coral reef habitat south of the GBR, the strengthening EAC can transport larvae poleward over large areas of open ocean negating the need for ‘stepping‐stone’ reefs to facilitate connectivity. This elevates the risk of COTS establishing populations on subtropical and warm‐temperate coral reefs in New South Wales (NSW) (Morello et al. 2014; Sommer et al. 2025). Indeed, there have been several records of Western Pacific *Acanthaster* cf. *solaris* occurrences across subtropical coral reefs in NSW, including the offshore Lord Howe Island (31.5553° S, 159.0821° E) (Harriott 1995) and Elizabeth Reef (29.9417° S, 159.0625° E) and Middleton Reef (29.4722° S, 159.1194° E) in the Tasman Sea. Most of these are singular observations (Table 1), although there are reports of outbreaks of COTS at Elizabeth and Middleton Reefs in 1981, 1987, 1994 and 2006 (Choat et al. 2006). After a moderate‐sized aggregation was recorded on the southern GBR Swain Reef complex in 1985 and at Middleton and Elizabeth Reefs in 1987 (Table 1), surveys were also carried out at nearby Lord Howe Island which recorded eight individuals (DeVantier and Andrews 1987).

**TABLE 1:** Historic observations of COTS from subtropical reefs in NSW.

Date	Location	Notes	Observer	Source
Feb 1975	Lord Howe Island	Collected (AustMus)	Brown/Wyburn	ALA (2020)
Dec 1981	Elizabeth and Middleton Reef	> 100 ind.	AIMS	Hutchings (1992)
Jan 1985	Lord Howe Island	Collected (AustMus)	Hoggett/Vail	ALA (2020)
1986	Anemone Bay, North Solitary Island	Outbreak, 10–20 ind.	Moody, S	DeVantier and Andrews (1987)
Dec 1987	Middleton Reef	Collected × 6 (AustMus)	Berents, P	ALA (2020)
Dec 1987	Elizabeth Reef	Collected × 2 (AustMus)	Berents, P	ALA (2020)
Feb 1988	Bubble Cave, North Solitary Island	Feeding scar	Smith, SDA	Pers obs
May 1989	Anemone Bay, North Solitary Island	Feeding scar	Smith, SDA	Pers obs
Jan 1992	Bubble Cave, North Solitary Island	450 mm ind.	Smith, SDA	Pers obs
Dec 1999	Sink Holes, North Solitary Island	One ind.	Shaw, I	Pers obs
Nov 2002	Cleaner Station, SSI	Small ind.	Smith, SDA	Pers obs
Jan 2003	Mathers Mooring, North Solitary Island	One ind.	Edgar, R (Shaw, I)	Pers obs
Jul 2004	Anemone Bay, North Solitary Island	One ind.	Shaw, I	Pers obs
Jun 2018	Elbow Cave, SNI	One ind.	Shaw, I	Pers obs
Aug 2020	Anemone Bay, North Solitary Island	One ind.	Shaw, I	Pers obs
Nov 2020	Trail Mooring, North Solitary Island	One ind.	Vaughan, N	Pers obs
Nov 2021	Anemone Bay, North Solitary Island	One ind.	Shaw, I	Pers obs
Feb 2022	Anemone Bay, North Solitary Island	One ind.	Shaw, I	Pers obs
Apr 2024	Anemone Bay, North Solitary Island	One ind.	Shaw, I	Pers obs

The southernmost documented report of COTS in coastal eastern Australia was from the Solitary Islands, NSW (30.2052° S, 153.2671° E) in 1987 (DeVantier and Andrews 1987) (although there are anecdotal reports that predate this (NS, pers. comm.)). Subsequently, small numbers of COTS have been observed regularly at North Solitary Island (29.9294° S, 153.3915° E) over the past 37 years (Figure 1, Table 1). However, in November 2024, the first localised outbreak comprising a mixed aggregation of Western Pacific *Acanthaster* cf. *solaris* (see nomenclatural note in methods) and the lesser‐known deep water congener *Acanthaster brevispinus* Fisher, 1917 was observed at North Solitary Island, the latter for the first time (Sommer et al. 2025). Surveys revealed considerable feeding damage by both *Acanthaster* species to slow growing scleractinian corals, including subtropical endemics and high‐latitude specialists across a large part of the leeward reef of North Solitary Island (Sommer et al. 2025). This outbreak provided a unique opportunity to test hypotheses about range expansion dynamics to inform subsequent management options for this range expanding species. We predicted that larvae most likely arrived from populations upstream in the EAC (GBR) rather than from another location in the South Pacific where this species also occurs. Further, we also predicted that genetic diversity would be low, suggestive of a single founder event. We analysed partial mitochondrial DNA sequences to determine the origins of the North Solitary Island COTS outbreak, assessed genetic connectivity with potential source populations and evaluated the demographic history of this range‐edge population. Understanding genetic origins and connectivity patterns is important for understanding future outbreak risk and to aid development of appropriate management responses.

## 2 Methods

COTS (*n* = 30) were collected from 12 to 18 m on the leeward reef of North Solitary Island on 13 November 2024, 14–15 January 2025 and 23 July 2025 within a mixed species aggregation. Tube feet tissue samples were taken from each specimen (*n* = 30) and preserved in 100% denatured ethanol and stored at −80°C. Tube feet tissue samples collected from *A*. cf. *solaris* at One Tree Island (OTI) in 2014 and 2019 were also used as this location in the southern Great Barrier Reef (GBR) represents one of the closest sources of COTS propagules to the study region. In addition, COTS from OTI have not previously been genetically characterised.

Tissue samples were used for DNA extraction, where 40 μg of tube feet tissue were processed using a Qiagen Blood and Tissue DNA extraction kit following the manufacturer's instructions without variation. The concentration, purity and quality of extracted DNA were tested using a Nanodrop spectrophotometer and by standard gel electrophoresis. A polymerase chain reaction to generate amplicons of the partial mitochondrial *cytochrome C oxidase I* (COI) marker was run using primers developed by Vogler et al. (2008): F4734, 5′‐GCCTGAGCAGGAATGGTTGGAAC‐3′ and R5433, 5′‐CGTGGGATATCATTCCAAATCCTGG‐3′. This reaction was set‐up using the Invitrogen Hot Start x 2 master mix kit incorporating 1 μL of unpurified template DNA in 50 μL reactions following the manufacturer's instructions. Cycling conditions were set as per the master mix manufacturer's instructions for amplicons > 500 bp without variation. Amplification (~658 bp) was validated using standard gel electrophoresis. Dual‐direction Sanger sequencing was processed by the Australian Genome Research Facility, Sydney using 20 μL of unpurified amplicon at a concentration of 10 μg/μL per sample.

Sequencing was successful for 23 North Solitary Island, and eight One‐Tree Island samples and paired‐single direction reads as .*ab* files were imported into *Geneious Prime* 2023.2.1 (Kearse et al. 2012) for de novo assembly using strict settings. Assemblies were quality checked for accurate base‐calling, edited manually where necessary and converted to consensus sequences for alignment. In addition, COI sequences of Western Pacific COTS lineage (see *note*) from across its range were also retrieved from GenBank (Clark et al. 2016) (Table 2) and aligned with the newly generated sequences from North Solitary Island and One‐Tree Island samples in *Geneious* using the MAFFT alignment tool (Katoh and Standley 2013). MAFFT settings were set to default and allowed for directional adjustment. The resulting alignment was then inspected for errors and further quality checked by protein translation using the echinoderm mtDNA genetic code.

**TABLE 2:** Details of COI sequences developed in this study and uploaded to the European Nucleotide Archive (ENA) [marked as *] and those retrieved from GenBank.

Location	GenBank/ENA accessions (*N*)	Source
North Solitary Island, NSW, Australia	ERS24976941‐ERS24976953*(13); PX405618‐PX405627 (10)	This study
One‐Tree Island, QLD, Australia (2014)	PV931806, PV931807 (2)	This study
One‐Tree Island, QLD, Australia (2019)	PV931808‐PV931813 (6)	This study
Dampier, WA, Australia	FM174522‐FM174529 (8)	Vogler et al. (2008)
Gove, NT, Australia	FM174530‐FM174536 (7)	Vogler et al. (2008)
Guam	FM174500‐FM174507 (8)	Vogler et al. (2008)
Jakarta (Pulau Seribu), Indonesia	FM174537‐FM174544 (8)	Vogler et al. (2008)
Kermadec Islands, New Zealand	KF012825‐KF012828 (4)	Liggins et al. (2014)
Lizard Island, QLD, Australia	FM174514‐FM174521 (8)	Vogler et al. (2008)
	KF012825‐KF012826 (9)	Liggins et al. (2014)
Okinawa, Japan	FM174508‐FM174513 (6)	Vogler et al. (2008)
Philippines	FM177197‐FM177203 (7)	Vogler et al. (2008)
	OR654053‐OR654082 (30)	GenBank, direct submission.
Solomon Islands	KF012825‐KF012826 (3)	Liggins et al. (2014)
Vanuatu	FM177190‐FM177196 (7)	Vogler et al. (2008)

The alignment was exported in FASTA format for importation into the *R* package *geneHapR* (Zhang et al. 2023) for analysis and generation of a haplotype network and haplotype distribution map to allow visualisation of connectivity patterns. Population‐level diversity statistics were calculated using the FASTA alignment in the DNA Sequence Polymorphism v6.12.03 (DnaSP) software package (Rozas et al. 2017). Demographic history of eastern Australian populations (North Solitary Island, One‐Tree Island and Lizard Island) was assessed using mismatch distribution analysis also in DnaSP. Observed pairwise nucleotide differences were compared to expected distributions under a constant population size model, with goodness‐of‐fit evaluated using a raggedness index. Genetic differentiation among populations was assessed using PERMANOVA (*adonis2 function*) in the *R* package *vegan* v2.6 based on Kimura 2‐parameter genetic distances from aligned COI sequences. Overall population structure was tested with 999 permutations, and pairwise comparisons were conducted with Benjamini‐Hochberg FDR correction for multiple testing. Plot outputs generated by the *geneHapR* package were imported into Inkscape (Inkscape Project 2020) to develop graphic figures.

*Taxonomic note*: We follow Vogler et al. (2008) and use informal, open taxonomy in reference to the COTS species studied here, *Acanthaster* cf. *solaris*. Until 2008, COTS were thought to be a single, broadly‐distributed species, *Acanthaster planci* (Linnaeus, 1758), but recent work by Leiva et al. (2025) confirmed polyphyly among the broad Pacific *Acanthaster* cf. *solaris* discussed in Vogler et al. 2008 and found that this species complex actually comprises three morphologically cryptic lineages, namely *A*. cf. *solaris* (Hawaii), *A*. cf. *solaris* (French Polynesia) and *A*. cf. *solaris* (Western Pacific), wherein limits for these lineages were defined by geography.

Following Leiva et al. (2025), we herein restrict the haplotype data used in the present study to those within the geographic bounds identified for the Western Pacific *A*. cf. *solaris* lineage: eastern limit = Fiji (178° E) and the Marshall Islands (178° E) and west to Vietnam, Borneo and Java (Jakarta) and the north western coast of Western Australia.

### 2.1 Limitations of COI‐Based Inference

Genetic inferences based on short fragments of the mitochondrial COI gene primarily reflect maternal lineages among a few loci. As a result, mitochondrial markers may differ from genome‐wide patterns due to their smaller effective population size, lineage sorting and potential selection on the mitogenome. In *A*. cf. *solaris*, mitogenome‐based analyses have suggested widespread connectivity across the Pacific (Yasuda 2018), whereas recent genome‐wide studies using single nucleotide polymorphisms have recovered clearer population structure and largely local outbreak origins (Leiva et al. 2025). The results generated in the present study should be interpreted as describing COI‐based mitochondrial connectivity and demographic history, whereas future genome‐scale datasets ought to be used to fully resolve the nuclear genomic context of outbreak populations in the subtropics.

## 3 Results

### 3.1 Population Structure

Analysis of 133 individual Western Pacific *Acanthaster* cf. *solaris* COI sequences from across 11 Pacific populations identified 30 haplotypes distributed across 590 loci of which 28 were polymorphic (Table 3). Overall genetic diversity across the study area was high (Hd ± SD = 0.74 ± 0.036), with moderate nucleotide diversity (π ± SD = 0.003 ± 0.00028). Global neutrality tests revealed significant signals of demographic expansion (Tajima's *D* = −1.90, *p* < 0.05; Fu's Fs = −26.5).

**TABLE 3:** Summary diversity statistics for *Acanthaster* cf. *solaris* populations in the Pacific from DnaSP analysis: *H*, number of haplotypes; Hd (SD), haplotype diversity (standard deviation); *N*, number of samples; π (SD), nucleotide diversity.

Location	*n*	*H*	Hd ± SD	π ± SD	Tajima's *D*	Fu's *F*
North Solitary Island, NSW	23	7	0.72 (0.07)	0.003 (0.0004)	−1.18	−1.69
One‐Tree Island, QLD	8	2	0.57 (0.12)	0.002 (0.0005)	1.79	2.21
Lizard Island, QLD	19	7	0.63 (0.07)	0.003 (0.0004)	−0.71	1.05
Vanuatu	7	5	0.90 (0.01)	0.007 (0.0013)	0.35	−0.18
Solomon Islands	3	2	0.67 (0.31)	0.002 (0.0010)	NA	NA
Dampier, WA	8	2	0.25 (0.18)	0.004 (0.0003)	−1.05	−0.18
Gove, NT	7	3	0.52 (0.20)	0.010 (0.0004)	−1.24	−0.92
Guam	8	5	0.86 (0.01)	0.003 (0.0007)	−0.17	−1.31
Jakarta, ID	8	4	0.64 (0.18)	0.002 (0.0007)	−1.53	−1.24
Okinawa JP	6	3	0.73 (0.15)	0.004 (0.0003)	−0.05	−0.43
Philippines	36	15	0.82 (0.06)	0.003 (0.0004)	−1.54	−10.70
All	133	30	0.74 (0.036)	0.003 (0.00028)	−1.90*	−26.50

* *p* < 0.05.

There was moderately high haplotype diversity at the subtropical North Solitary Island outbreak site (Hd = 0.719 ± 0.07) and moderate nucleotide diversity (π = 0.003 ± 0.0004) based on the presence of nine polymorphic loci and seven haplotypes. Negative but non‐significant (*p* > 0.05), Tajima's *D* (−1.18) and Fu's Fs (−1.69) are consistent with a recent population expansion to North Solitary Island through an earlier colonisation event. The diversity measures at North Solitary Island exceeded several nearby tropical populations, including Lizard Island (Hd = 0.63), One‐Tree Island (Hd = 0.57), Solomon Islands (Hd = 0.67) and Gove (Hd = 0.52) but was less diverse than Vanuatu (Hd = 0.90). Population neutrality metrics varied between positive and negative (positive values may indicate population subdivision, balancing selection or population contraction [loss of rare haplotypes]) in eastern Australian and Coral Sea populations: One‐Tree Island (*D* = 1.79, Fs = 2.21), Lizard Island (*D* = −0.71, Fs = 1.05) and Vanuatu (*D* = 0.35, Fs = −0.18).

Further into the Western Pacific, the Philippines exhibited the highest haplotype richness (*H* = 15), with high haplotype diversity (Hd = 0.82 ± 0.06), and moderate nucleotide diversity (π = 0.0030 ± 0.0004). Here, demographic expansion is apparent with a strongly negative but non‐significant Fs (−10.7, *p* > 0.05). Nearby populations from Guam (*n* = 8) and Okinawa (*n* = 6) also exhibited high haplotype diversity (Hd = 0.86 and 0.73, respectively) with moderate nucleotide variation (π = 0.0031 ± 0.0007 and 0.0036 ± 0.0003, respectively). The Indian Ocean boundary populations, Jakarta (Hd = 0.64) and Dampier (Hd = 0.25) exhibited lower diversity and fewer polymorphic loci, but negative measures of neutrality (*D* = −1.53 + −1.05 and Fs = −1.24 + −0.18, respectively) indicate recent expansion.

PERMANOVA revealed significant genetic structure among populations (*F* = 4.33, *R* ^2^ = 0.262, *p* < 0.05). Pairwise analysis indicate that North Solitary Island was not significantly different from Lizard Island (*F* = 0.156, *p* > 0.05), One‐Tree Island (*F* = 0.447, *p* > 0.05) or the Solomon Islands (*F* = −0.036, *p* > 0.05), suggesting high mitochondrial connectivity among eastern Australian and some Coral Sea populations. However, there was significant differentiation between North Solitary Island and the Philippines (*F* = 9.28, *p* < 0.05) and Vanuatu (*F* = 10.29, *p* < 0.05) (Table 5).

### 3.2 Population Connectivity

**FIGURE 2:**
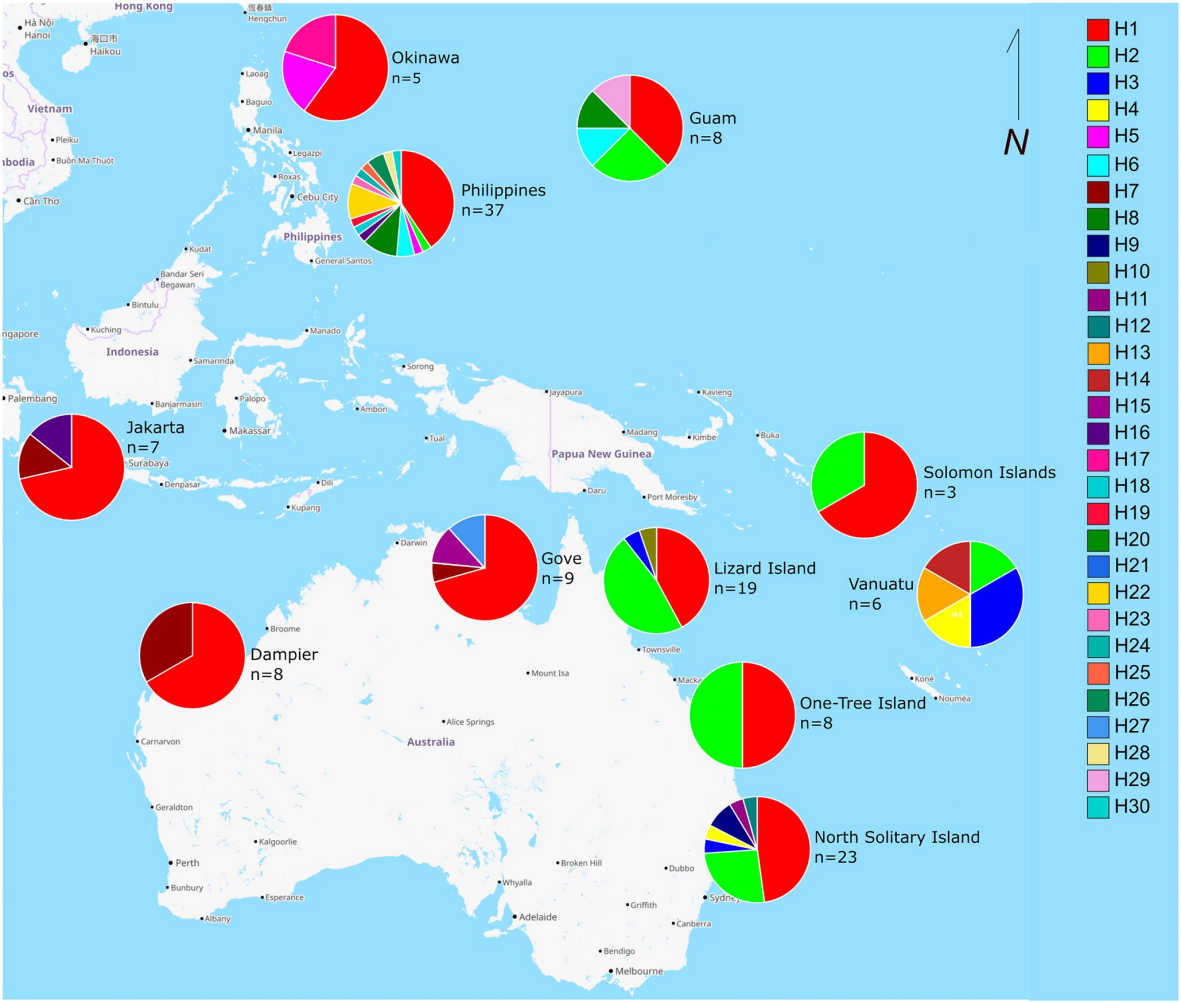
Map of haplotype distribution from COI sequence data for Western Pacific *Acanthaster* cf. *solaris*.

Haplotype mapping revealed clear patterns of mitochondrial genetic connectivity among populations (Figure 2, Table 3). Of the 30 identified haplotypes, 14 were non‐unique (shared across more than one population) with several of those shared broadly among geographically distant populations.

Two highly dominant haplotypes, H1 and H2, showed extensive geographic distribution (Table 4). Haplotype H1 was present in all but one of the sampled populations (Vanuatu) and accounted for 47% (63/133) of all sequences, with a distribution extending from North Solitary Island, One Tree Island and Lizard Island on the GBR through to the north‐western populations in the Philippines, Okinawa and Guam. Its broad distribution and high frequency suggest that H1 is likely to be an ancestral or founder haplotype. Haplotype H2, comprising 18% (24/133) of sequences, was also widespread, but restricted to the eastern part of the species' range, found across seven populations including North Solitary Island, One Tree Island, Lizard Island, Solomon Islands, Vanuatu and to the north at Guam and the Philippines.

**TABLE 4:** Distribution of Western Pacific *Acanthaster* cf. *solaris* haplotypes.

Population	Haplotype																														
	H1	H2	H3	H4	H5	H6	H7	H8	H9	H10	H11	H12	H13	H14	H15	H16	H17	H18	H19	H20	H21	H22	H23	H24	H25	H26	H27	H28	H29	H30	Total
North Solitary Island	11	6	1	1					2		1	1																			23
One‐Tree Island	4	4																													8
Lizard Island	8	9	1							1																					19
Vanuatu		1	2	1									1	1																	6
Solomon Islands	2	1																													3
Dampier/Gove	12						1								1						1						2				17
Jakarta	5						1									1															7
Philippines	15	1			1	2		4								1		1	1			4	1	1	1	2		1		1	37
Okinawa	3				1												1														5
Guam	3	2				1														1									1		8
Total	63	24	4	2	2	3	2	4	2	1	1	1	1	1	1	2	1	1	1	1	1	4	1	1	1	2	2	1	1	1	133

**TABLE 5:** Pairwise PERMANOVA analysis for Western Pacific *Acanthaster* cf. *solaris* populations based on mitochondrial COI sequences.

Population	North solitary (23)	One‐tree (8)	Lizard (19)	Vanuatu (7)	Solomon (3)	Philippines (36)	Okinawa (6)	Guam (8)	Jakarta (8)	Gove (7)	Dampier (8)
North Solitary	—	0.604	0.758	**0.005**	0.995	**0.001**	0.059	0.450	0.102	0.131	0.186
One‐Tree	0.447	—	0.887	**0.022**	1.000	**0.009**	**0.031**	0.852	0.056	**0.033**	**0.041**
Lizard	0.156	0.147	—	**0.010**	1.000	**0.001**	**0.028**	0.677	0.121	0.121	0.138
Vanuatu	10.29	6.67	9.54	—	0.185	**0.001**	0.084	**0.023**	0.065	0.078	0.083
Solomon	−0.036	0.205	−0.048	1.91	—	0.344	0.194	1.000	0.578	0.158	0.203
Philippines	9.28	6.11	9.70	15.75	1.23	—	0.166	**0.005**	0.977	0.852	0.923
Okinawa	3.31	7.70	3.65	3.66	2.90	1.70	—	0.075	0.125	0.152	0.393
Guam	0.879	0.137	0.503	6.56	0.007	5.73	3.72	—	0.105	0.091	0.157
Jakarta	2.40	4.59	2.36	4.52	1.18	−0.357	2.05	2.33	—	0.615	0.901
Gove	2.55	7.63	2.92	3.49	2.97	0.181	2.31	3.18	0.867	—	0.801
Dampier	1.90	7.99	2.28	3.35	3.47	−0.020	1.87	2.71	0.593	1.47	—

*Note:* Below diagonal: *F*‐statistic values. Above diagonal: *p*‐values (bold indicates *p* < 0.05 after Benjamini‐Hochberg correction). Sample sizes shown in parentheses.

**TABLE 6:** Mismatch distribution statistics for eastern Australian *Acanthaster* cf. *solaris* populations.

Population	*n*	*τ* (tau)	Raggedness
North Solitary Island	23	0.859	0.0982
One‐Tree Island	8	1.143	0.8367
Lizard Island	19	0.611	0.3897

North Solitary Island was dominated by the cosmopolitan H1 (48%) and H2 (26%) haplotypes but also contained regionally restricted haplotypes such as H4 (found only at North Solitary Island and Vanuatu) and H3 (found only at North Solitary Island, Lizard Island and Vanuatu) and also three unique haplotypes (H9, H11 and H12) that were not found upstream at One Tree Island, Lizard Island or elsewhere in the Coral Sea populations.

With 15 haplotypes, the Philippines was highly diverse, with many occurring as singletons or at low frequencies (most likely due to high sample numbers). Several of these rare haplotypes (H8, H18, H19, H22–26, H28, H30) were unique to the Philippines suggesting that this population may be a genetic source for the lineage. Other Western Pacific peripheral populations also harboured unique haplotypes Dampier/Gove (H15, H21, H27); Vanuatu (H13, H14); Okinawa (H17), Guam (29) (Table 4).

**FIGURE 3:**
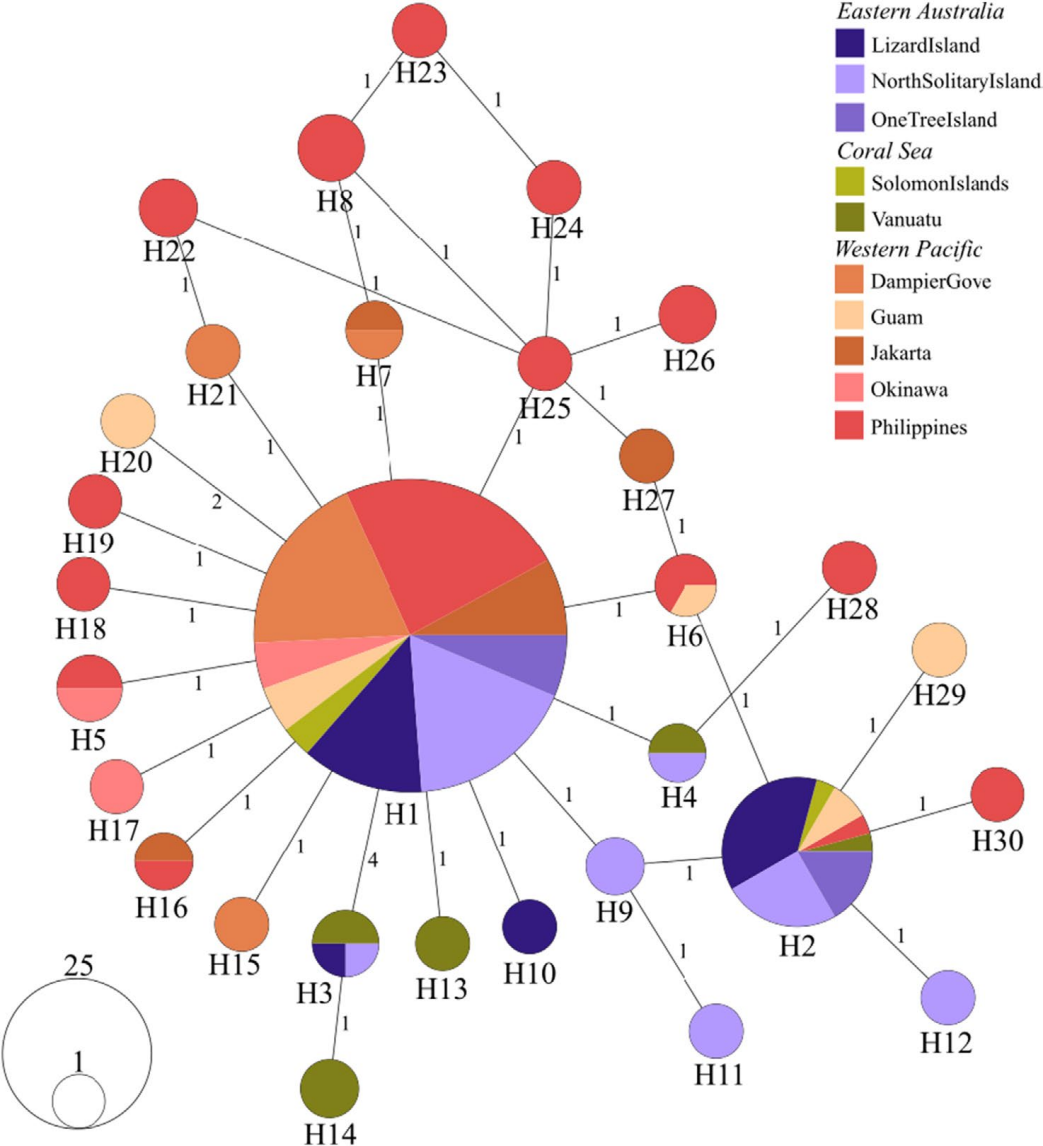
Neighbour‐joining haplotype network for *Acanthaster* cf. *solaris* across the Western Pacific based on COI sequence data.

Haplotype network visualisation displayed a star‐like pattern with multiple haplotypes radiating from two central nodes, indicating recent population expansion from the likely ancestral haplotypes, H1 & H2. The Philippines population was highly diverse with many unique and shared haplotypes that connected to most other populations. Haplotype sharing among distant populations (e.g., long‐distance connections of H1 and H2 from the Philippines to North Solitary Island) indicates recent broadscale gene flow or retention of ancestral polymorphisms across the species' range. This network topology, characterised by short branch lengths between most haplotypes, is consistent with recent demographic expansion following a population bottleneck, which aligns with the known outbreak dynamics and dispersal patterns of *A*. cf. *solaris* across coral reef systems in the Indo‐Pacific (Figure 3).

Eastern Australian and Coral Sea populations exhibited separation from northern and western populations by at least one mutated loci and connections with the eastern H2 haplotype suggests minor geographic subdivision. These eastern populations showed evidence of emerging regional clustering wherein the haplotype with the longest chain of nucleotide variation from the ‘ancestral’ H1, H14 (which is a total of 5 variant loci) was found at Vanuatu, itself derived from the shared H3 haplotype (4 mutations from H1) which is restricted to Vanuatu, Lizard Island and North Solitary Island.

### 3.3 Population Dynamics in Eastern Australian Populations

**FIGURE 4:**
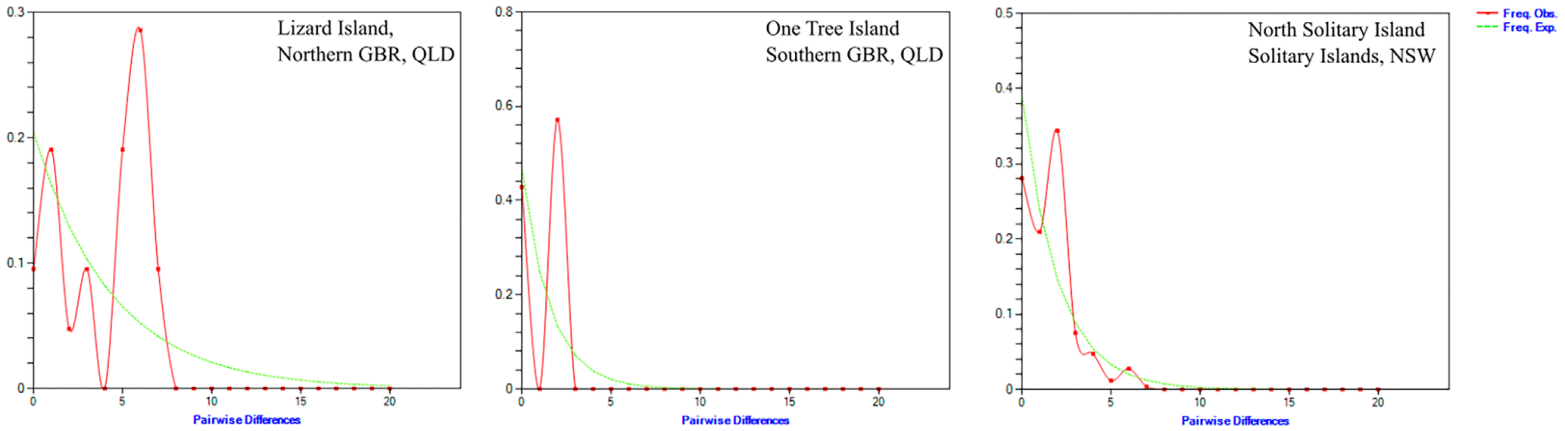
Pairwise population mismatch distribution plots for Lizard Island, QLD, One‐Tree Island, QLD and North Solitary Island, NSW. Green = expected frequency of pairwise differences under a constant population size model, Red = observed frequency of pairwise differences under a constant population size model.

An analysis of population size dynamics for the eastern Australian populations: Lizard Island, QLD, One‐Tree Island, QLD and North Solitary Island, NSW, was carried out using pairwise differences (mismatch distribution) and a constant population size model to determine expected values. The North Solitary Island analysis recovered a unimodal (single‐peak) distribution with good fit between observed and expected frequencies (Figure 4). This pattern strongly suggests a recent demographic expansion (rapid recruitment from a small effective population size). The peak at two differences suggests that most individuals are very closely related, sharing recent common ancestors and a smooth decaying curve (rather than a ragged, multimodal distribution). This supports the notion of a single expansion event rather than a constant population size or multiple expansion/contractions. The close fit to the model further suggests that expansion was relatively recent, rapid rather than gradual. The One‐Tree Island population exhibited a similar unimodal distribution suggesting a recent expansion but with a poorer fit to the model (Table 6). Conversely the population at Lizard Island on the northern GBR exhibited a multimodal pattern with a major peak at six differences and smaller peaks at lower values. This suggests a longer, more complex demographic history of *A*. cf. *solaris* at Lizard Island compared with North Solitary Island with putative multiple lineages and a history of secondary contact with previously separate populations.

## 4 Discussion

Understanding the origins and frequency of outbreaks of COTS on subtropical reefs are key for informing management strategies under climate change. Although small numbers of COTS have regularly been observed in the subtropical Solitary Islands for some 30+ years, the first physically observed outbreak of COTS in 2024/2025 (Sommer et al. 2025) provided the opportunity to determine the origins of these individuals to inform subsequent management and control programs. Although we predicted that the range edge North Solitary Island population would have lower genetic diversity compared to core populations due to founder effects, genetic drift and isolation (Eckert et al. 2008), we instead found moderate levels of COI genetic diversity and shared haplotypes with proximal Pacific populations which suggest that the source of this outbreak may be from more than one dispersal event. Indeed, negative neutrality statistics and a unimodal peak for pairwise differences in the population mismatch distribution plot (Figure 4) suggest that the population is an early‐stage colonisation event or undergoing recent demographic expansion. Among this outbreak population, the presence of the cosmopolitan [H1] and widespread eastern haplotype [H2] in addition to shared regional haplotypes such as H4 (also found at Vanuatu) and H3 (also found at Lizard Island and Vanuatu) indicate larval connectivity through dispersal in the EAC from the GBR (Condie et al. 2012; James et al. 2002) to the study region. In northern NSW, recruitment is likely to be through episodic larval dispersal mediated by ocean currents following the broader trends of ‘tropicalisation’ of temperate marine ecosystems in southeastern Australia (Vergés et al. 2014).

Identifying the genetic structure and connectivity of peripheral outbreak populations of COTS at North Solitary Island is critical for understanding the potential for further outbreaks. PERMANOVA analysis confirmed high mitochondrial connectivity between North Solitary Island and GBR populations, with no significant differentiation from Lizard Island or One Tree Island despite the ~1500 km distance. This lack of differentiation, combined with the presence of shared cosmopolitan haplotypes (H1, H2) and regionally restricted haplotypes (H3, H4), supports the notion of recurrent larval connectivity through the East Australian Current from tropical source populations. The significant differentiation between North Solitary Island and distant populations suggests that although populations such as those in the Philippines serve as a broader regional source of genetic diversity, direct larval recruitment to North Solitary Island most likely occurs primarily from the GBR rather than distant populations.

Connectivity patterns with populations to the north and north‐east could inform the risk of outbreaks by identifying source populations and high‐risk recruitment zones for monitoring (Babcock et al. 2020). Connectivity patterns also inform whether outbreak risk is likely to increase at North Solitary Island in the future or whether the outbreak was an unusual or unique event. The evidence of both moderate COI genetic diversity and connectivity with reefs in the northern (Lizard Island exhibited regional connectivity and a more complex demographic history than both One Tree Island and North Solitary Island) and southern GBR and east into the Coral Sea (Hock et al. 2017) suggests that establishing a regular surveillance program for COTS outbreaks at the small but important subtropical coral‐dominated reefs in the Solitary Islands, as well as other coral‐dominated reefs in subtropical eastern Australia, would be important. Moreover, the development of management strategies and policy for interventions are critical to inform the actions taken when outbreaks occur. While larval dispersal and recruitment into subtropical reefs cannot be stopped, control programs may be implemented to slow population expansion on subtropical reefs and subsequent damage to endemic and subtropical corals (Sommer et al. 2025).

### 4.1 General Genetic Patterns Across the Western Pacific

Although a broad‐scale study of genetic diversity and connectivity among *A*. cf. *solaris* in the Pacific was carried out by Vogler et al. (2013) and Leiva et al. (2025), there has been no analysis using the mtDNA COI marker which is useful for identifying long‐term phylogeographic patterns (Avise et al. 1987) as in many studies (Fernando et al. 2020; Petit‐Marty et al. 2021; Hebert et al. 2003). Using these data, we reveal a central genetic hub around the central tropical Western Pacific (the Philippines) which is characterised by long‐term stability and may have undergone secondary (repeat) contact among divergent lineages. Although global diversity metrics indicate that most populations are neither genetically homogeneous nor totally inbred, the Philippines exhibited the highest haplotype richness, which may partly reflect its larger sample size. However, standardised haplotype diversity (Hd = 0.82) and its biogeographic position support the notion that this population has disproportionately contributed to mitochondrial (COI) genetic diversity across Western Pacific distribution of *A*. cf. *solari*s.

The observed star‐like haplotype network topology and negative Tajima's *D* and Fu's Fs values suggest historic demographic expansion following post‐glacial range shifts (last glacial maximum was ~12 k y/a) (Grant and Bowen 1998), a common pattern among broadcast‐spawning echinoderms (Garcia‐Cisneros et al. 2016; Maltagliati et al. 2010). The site of contemporary coral communities in the subtropical Solitary Islands in eastern Australia was continental during the last glacial maxima, with the paleo coastline at that time well to the east (Brooke et al. 2017; Lewis et al. 2013; Williams et al. 2018). It is probable that central populations like the Philippines acted as historical refugia during glaciation and were the source for recolonisation across the Western Pacific (Crandall et al. 2012) as sea levels subsequently rose.

COTS have natural predators, for example Triton Snails, *Charonia tritonis* (Linnaeus, 1758), Humphead Wrasse, *Cheilinus undulatus* Rüppell, 1835 and some tetraodontiform fishes (Chesher 1969; Laxton 1971; Ormond et al. 1990) and environmental factors (e.g., low nutrient load) are thought to regulate COTS populations on pristine reefs (Birkeland and Lucas 1990). However, loss of predators or alteration of environmental conditions can result in sudden and significant population booms (Sweatman 2008). It is unlikely however that the 2024/2025 North Solitary Island outbreak was solely in response to reduced predation pressure (given that North Solitary Island sits well within a large marine protected area) even though there may be a mismatch in the poleward range shift of predators which may partially facilitate outbreaks. With the effects of warming, it is probable that the outbreak was a response to favourable environmental conditions, that is a 2°C increase in water temperature and, with adequate food, larval development time may be reduced by 30% and probability of survival increases by 240% (Lamare et al. 2014). In the juvenile stage, COTS are herbivores, and in the absence of coral prey, they may remain so, hidden in the reef infrastructure poised to transition to corallivory should prey become available (Deaker et al. 2020; Wolfe and Byrne 2024). Their high fecundity and capacity to persist as herbivorous juveniles means that COTS populations may regenerate rapidly after population crashes or control efforts, posing a persistent threat to coral communities (Pratchett et al. 2017; Wolfe and Byrne 2024). The ability of *A*. cf. *solaris* to persist as herbivorous juveniles (at least 6.5 years) may have facilitated the first outbreak and also indicates the potential for a disconnect between initial recruitment and emergence on the reef as coral eaters (Deaker et al. 2020). This highlights the need for ongoing monitoring.

## 5 Conclusions

This study used opportunistic sampling of a rare, subtropical outbreak of Western Pacific *Acanthaster* cf. *solaris* at North Solitary Island, NSW to test the hypothesis that a population outbreak in a high latitude reef was driven by a single larval dispersal event from the GBR. Patterns of COI haplotype distribution indicate that source populations for individuals sampled at North Solitary Island in 2024/2025 were from areas further north with shared haplotypes with the GBR (Lizard Island and One Tree Island) and the northern and eastern Coral Sea (Vanuatu and the Solomon Islands). The levels of COI genetic diversity among individuals in the North Solitary Island outbreak suggest that it may not be a founder population (initial establishment) but rather an early but established population supported by ongoing recruitment, although genome‐wide data will be necessary to confirm the extent of self‐sustainability and local recruitment. With ongoing climate change in this region, management actions must consider the likely continued larval recruitment and potential for outbreaks in this area of high conservation value and develop appropriate monitoring, management and adaptation programs.

## Author Contributions

**Matt J. Nimbs:** conceptualization (lead), data curation (lead), formal analysis (lead), investigation (lead), methodology (lead), project administration (lead), resources (supporting), visualization (lead), writing – original draft (lead), writing – review and editing (lead). **Maria Byrne:** data curation (equal), formal analysis (supporting), investigation (equal), writing – original draft (supporting), writing – review and editing (supporting). **Steven J. Dalton:** data curation (supporting), investigation (supporting), methodology (supporting), resources (supporting), writing – original draft (supporting), writing – review and editing (supporting). **David Maguire:** conceptualization (supporting), data curation (supporting), investigation (supporting), methodology (supporting), writing – review and editing (supporting). **Hamish A. Malcolm:** conceptualization (supporting), data curation (equal), formal analysis (supporting), investigation (equal), methodology (lead), resources (supporting), writing – original draft (equal), writing – review and editing (supporting). **Nicole Strehling:** conceptualization (supporting), funding acquisition (lead), project administration (lead), resources (lead), writing – review and editing (supporting). **Brigitte Sommer:** conceptualization (supporting), data curation (equal), formal analysis (supporting), writing – original draft (supporting), writing – review and editing (supporting). **Melinda A. Coleman:** formal analysis (equal), funding acquisition (lead), investigation (equal), methodology (equal), project administration (lead), visualization (supporting), writing – original draft (supporting), writing – review and editing (equal).

## Funding

Funding for this study was provided by the New South Wales Marine Estate Management Strategy. S.B. was supported by an Australian Research Council Discovery Early Career Research Award (DE230100141).

## Conflicts of Interest

The authors declare no conflicts of interest.

## Data Availability

All data used in this study have been lodged in the publicly accessible GenBank and European Nucleotide Archive databases. COI sequences from specimens from North Solitary Island are available from GenBank (https://www.ncbi.nlm.nih.gov/nuccore) via the accessions PX405618‐PX405627 and ENA via accessions ERS24976941‐ERS24976953 and from One‐Tree Island in GenBank (https://www.ncbi.nlm.nih.gov/nuccore) via accessions PV931806‐PV931913.
